# Supplementary figures and images for: The integrated stress response remodels the microtubule-organizing center to clear unfolded proteins following proteotoxic stress
Source: eLife. 2022 Jun 27;11:e77780. doi: 10.7554/eLife.77780 (PMC9299849; doi:10.7554/eLife.77780)

Figure 1 - Source data 1 - Hurwitz et al

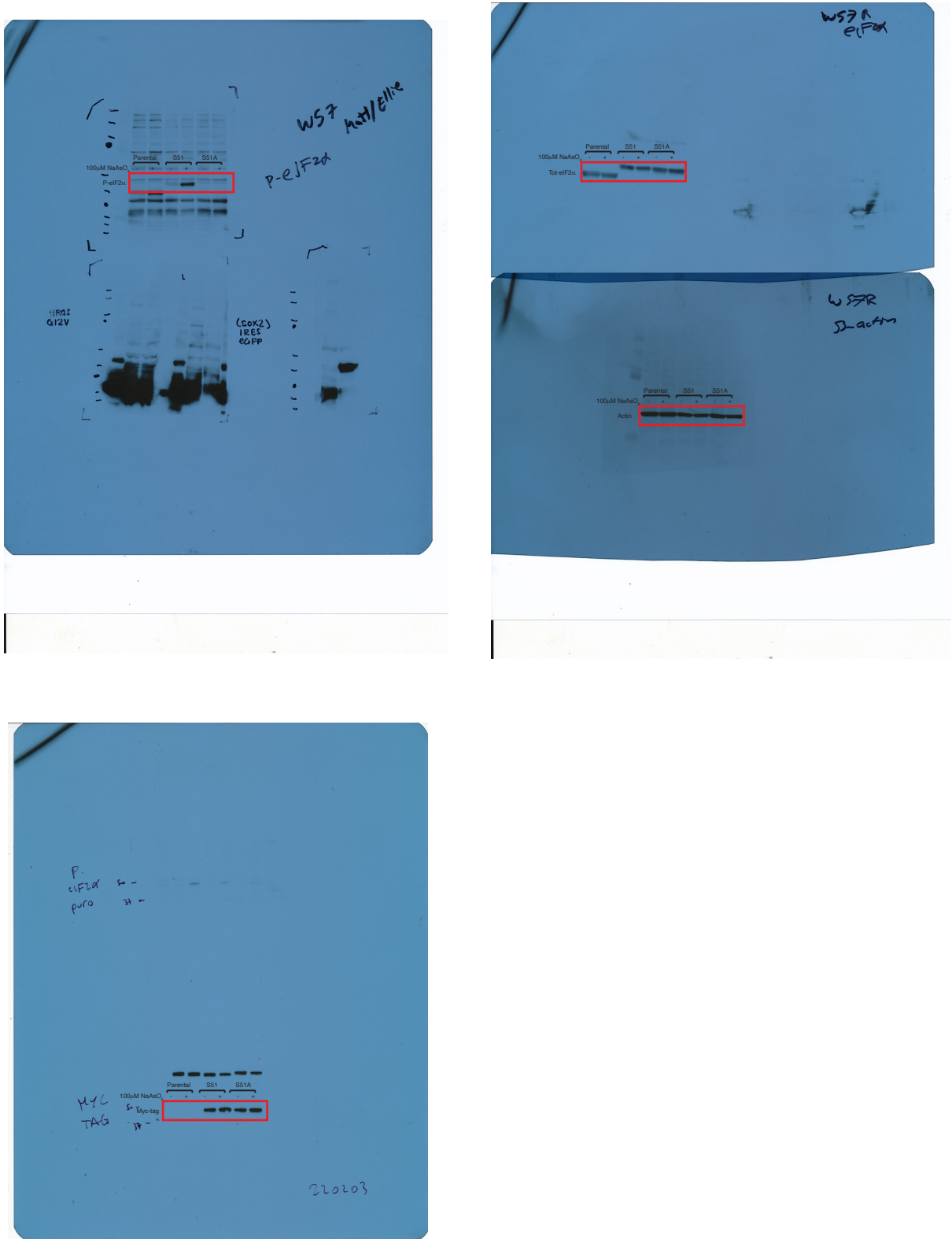

Supplement: Figure 1—source data 1. [file elife-77780-fig1-data1.pdf]

Figure 1 - Source data 2 - Hurwitz et al

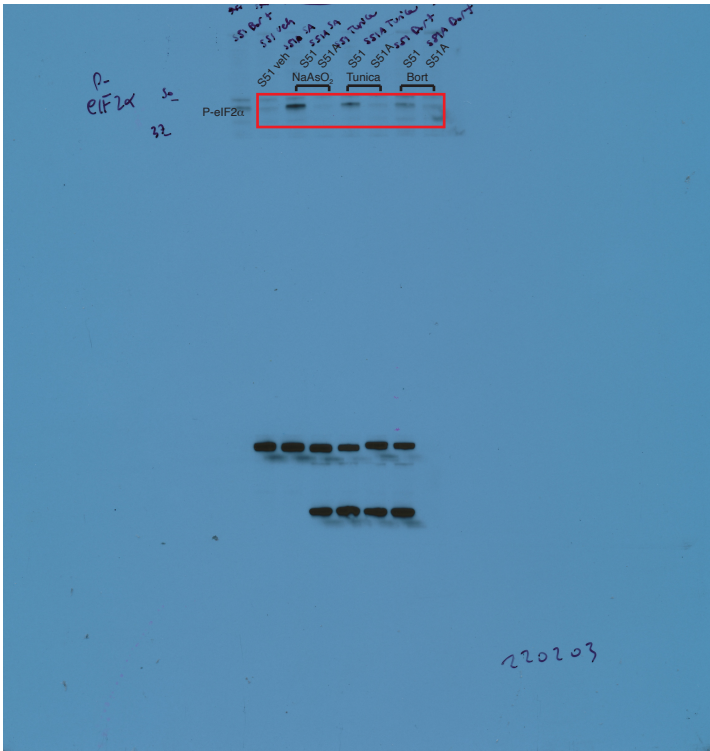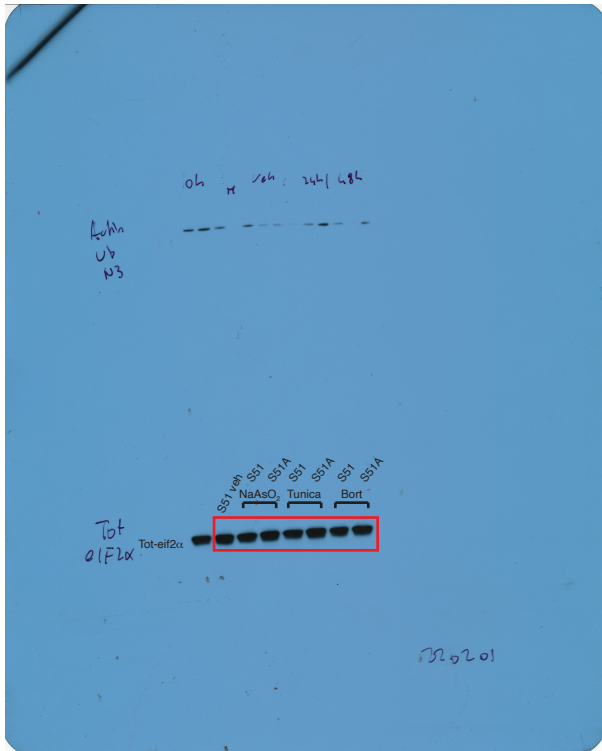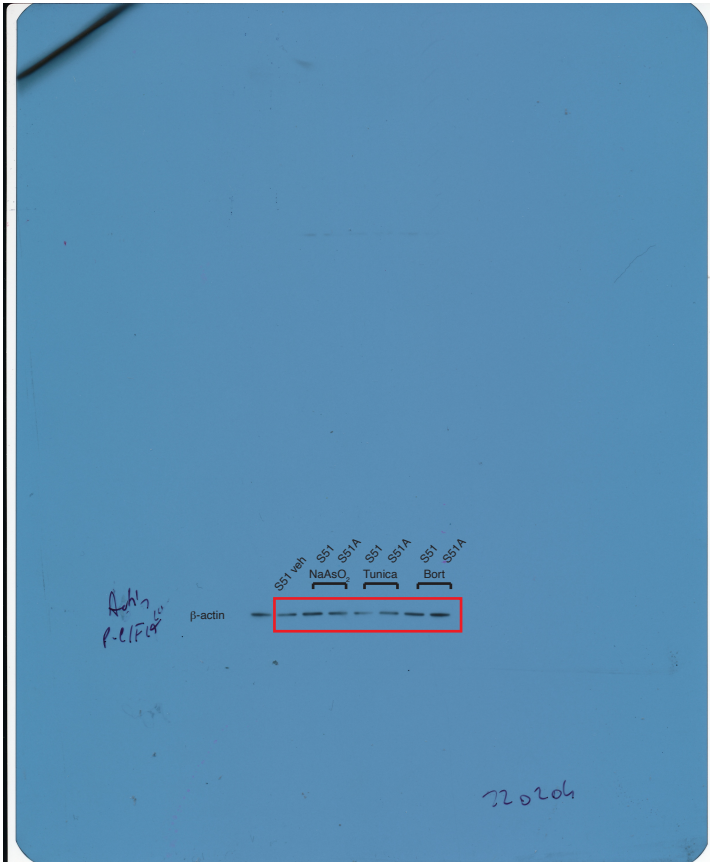

Supplement: Figure 1—source data 2. [file elife-77780-fig1-data2.pdf]

Figure 1 - Source data 3 - Hurwitz et al

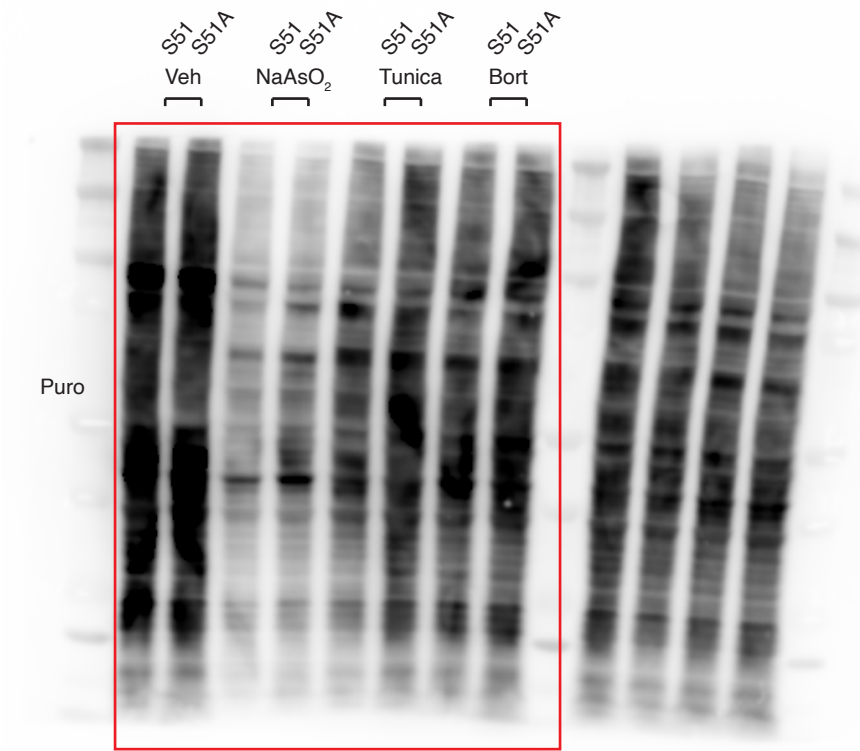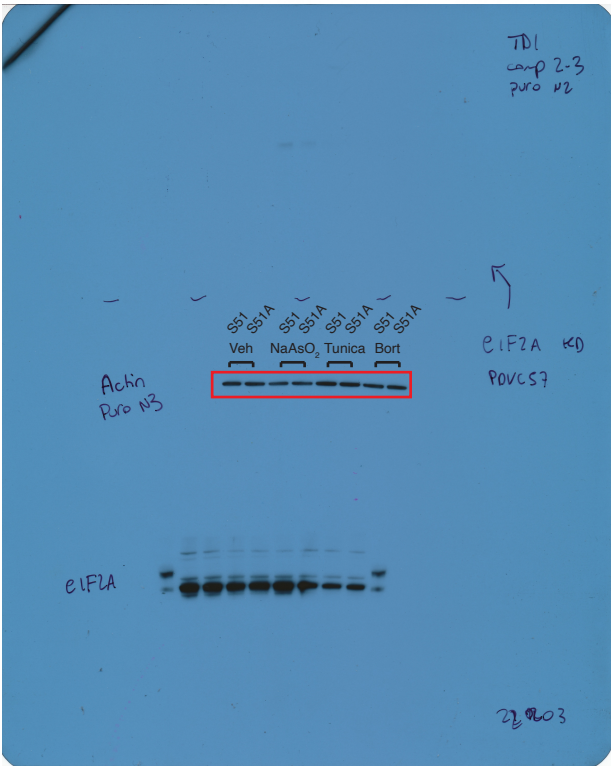

Supplement: Figure 1—figure supplement 1—source data 1. [file elife-77780-fig1-figsupp1-data1.pdf]

Figure 1 - Source data 4 - Hurwitz et al

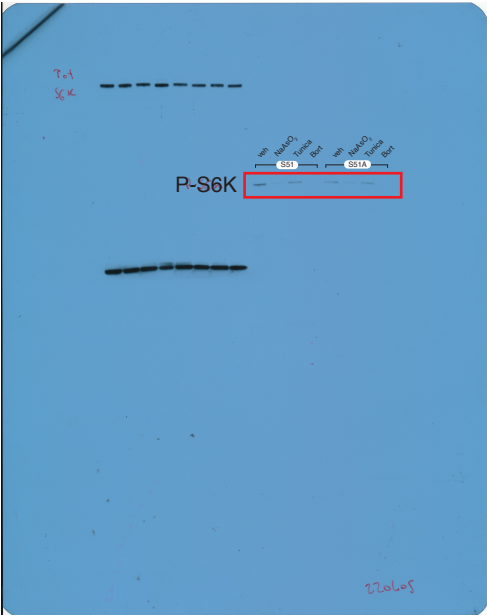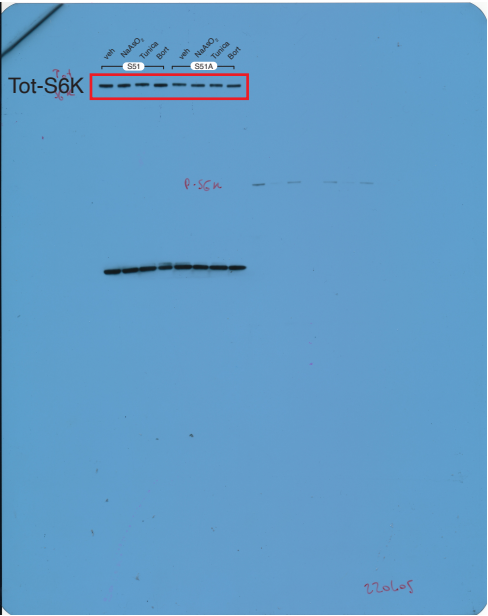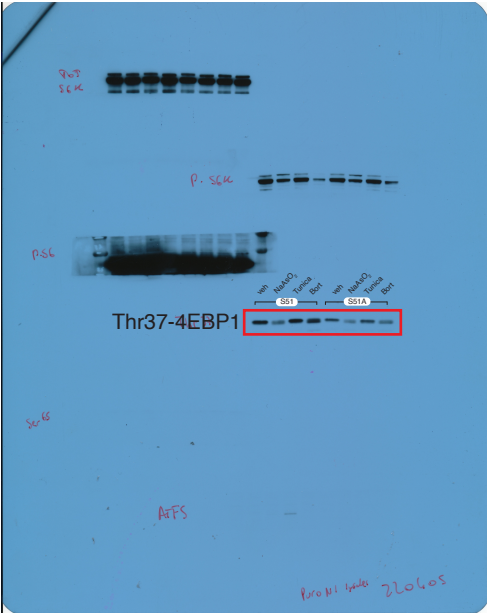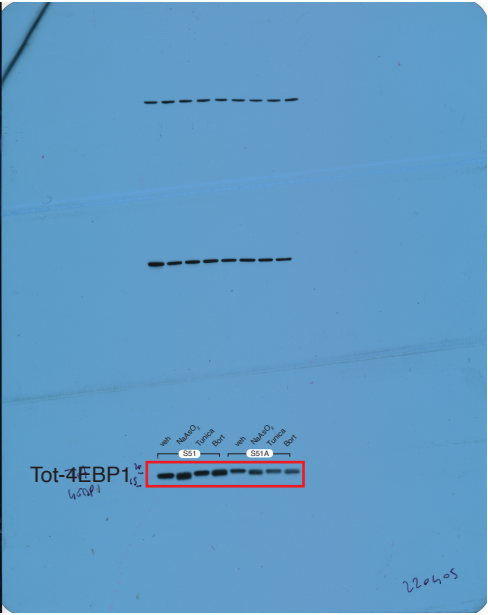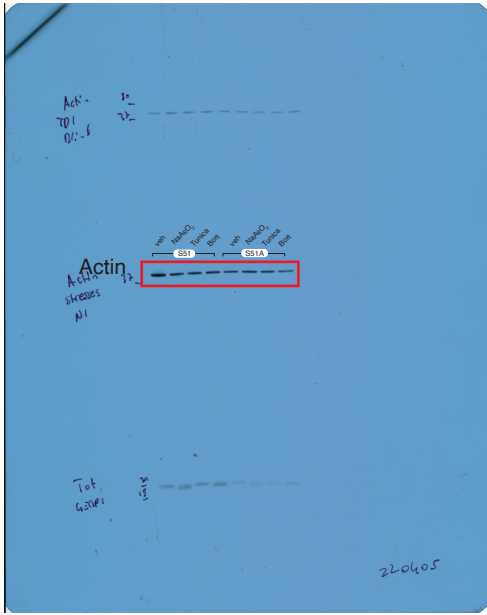

Supplement: Figure 1—figure supplement 1—source data 2. [file elife-77780-fig1-figsupp1-data2.pdf]

Figure 2 - Source data 3 - Hurwitz et al

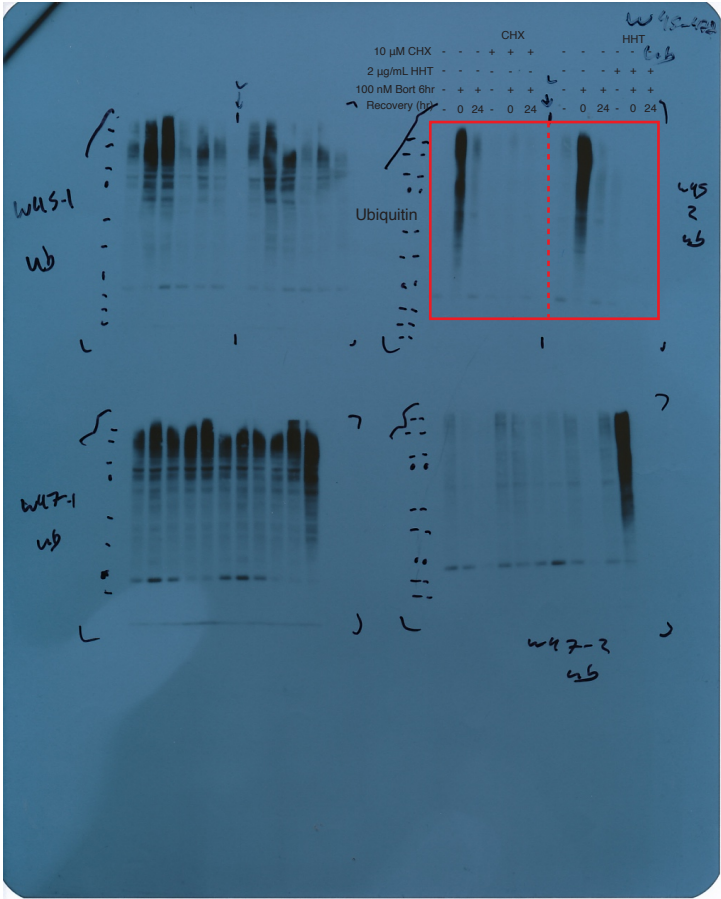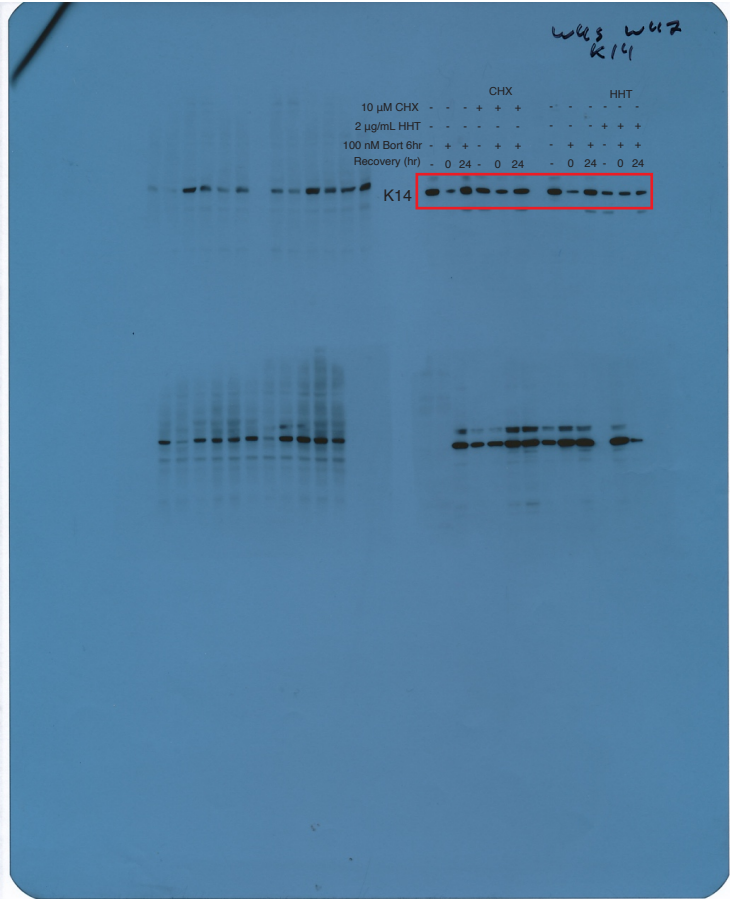

Supplement: Figure 2—figure supplement 3—source data 1. [file elife-77780-fig2-figsupp3-data1.pdf]

Figure 2 - Source data 4 - Hurwitz et al

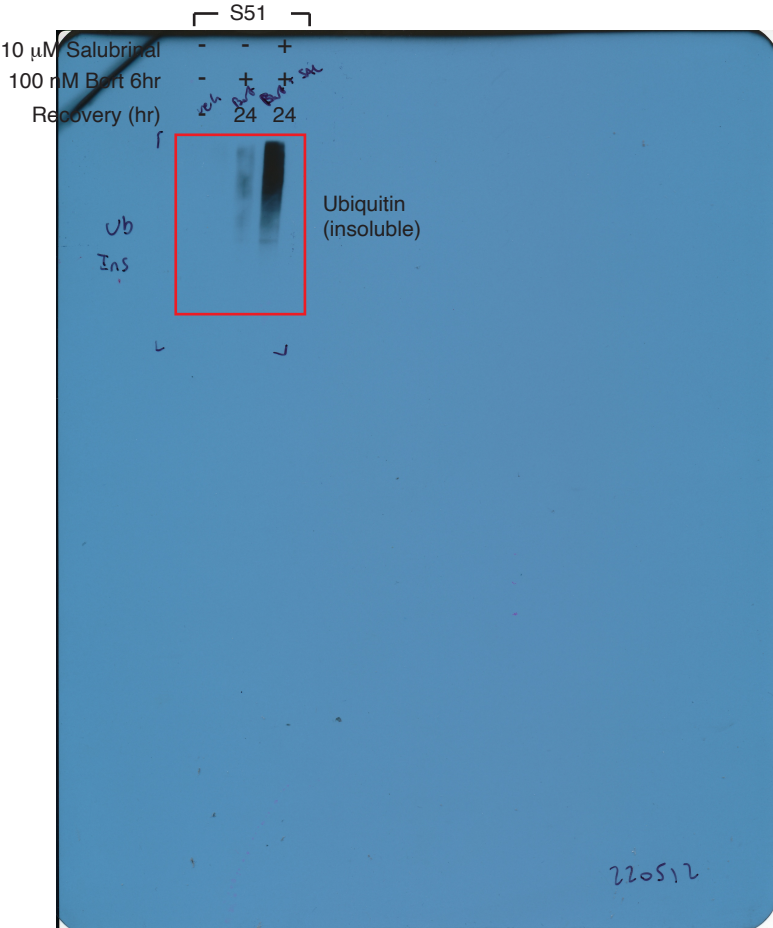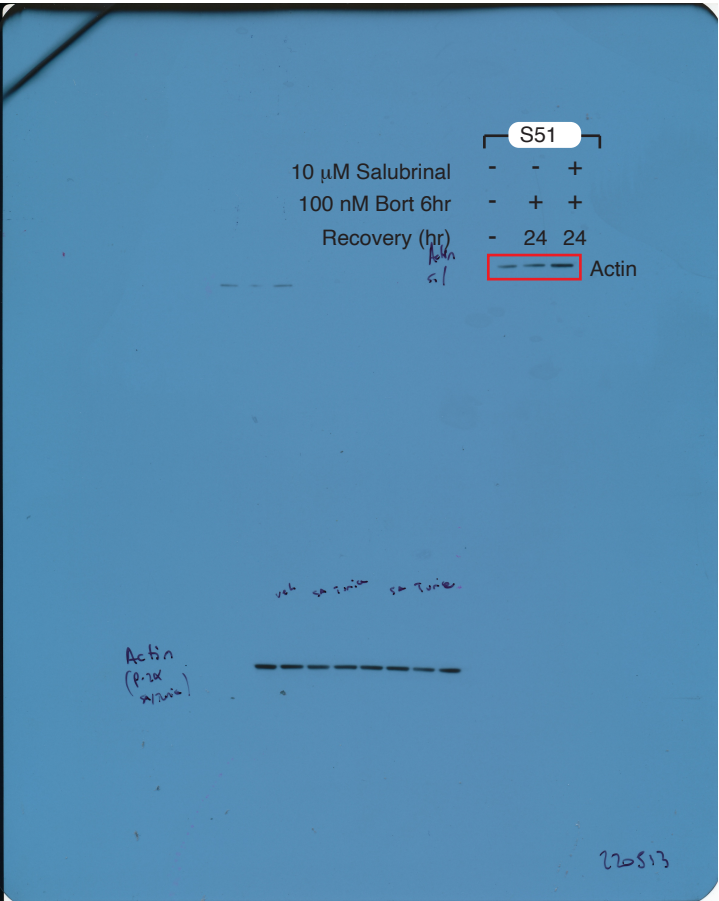

Supplement: Figure 2—figure supplement 3—source data 2. [file elife-77780-fig2-figsupp3-data2.pdf]

Figure 2 - Source data 5 - Hurwitz et al

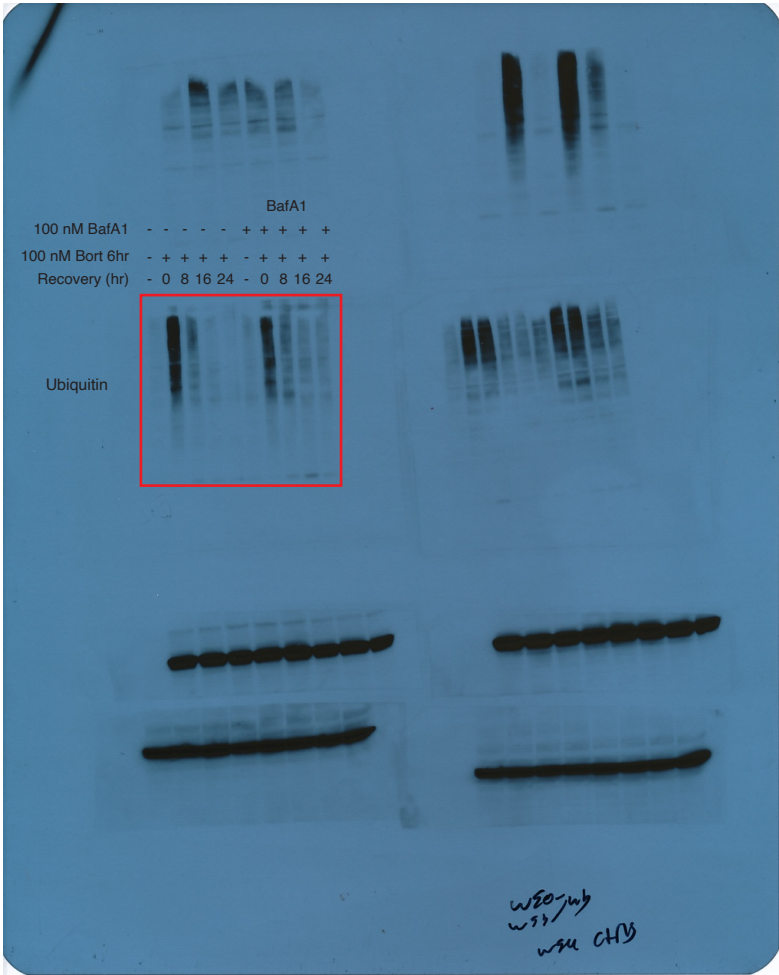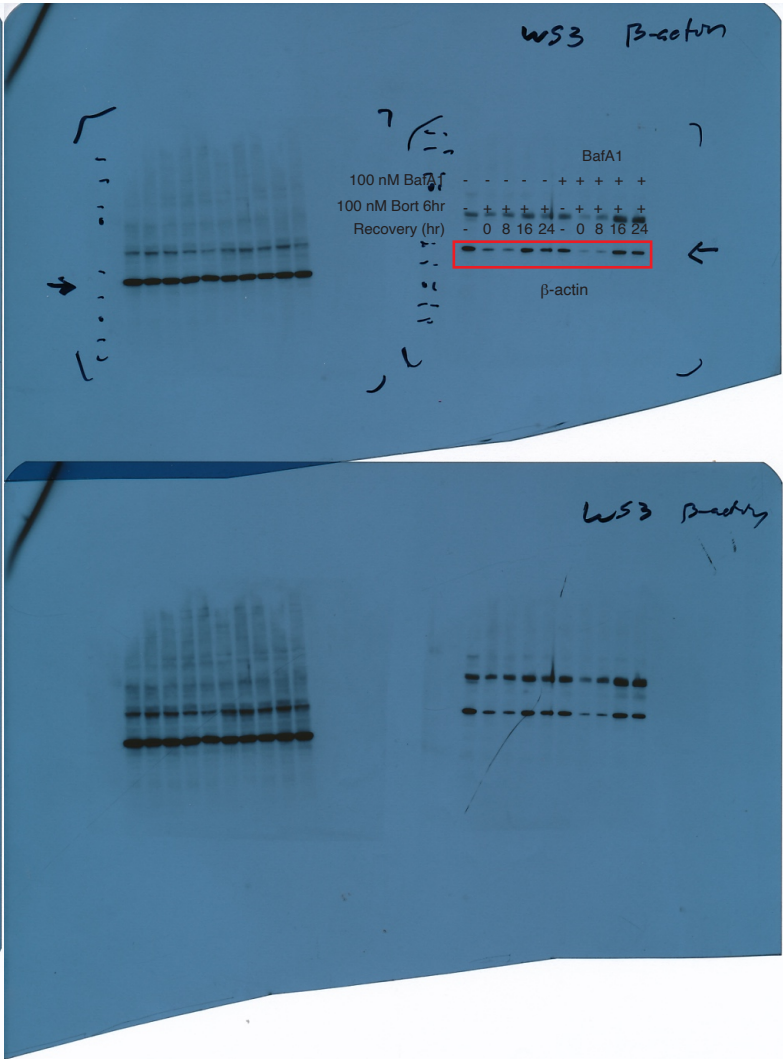

Supplement: Figure 2—figure supplement 3—source data 3. [file elife-77780-fig2-figsupp3-data3.pdf]

Figure 2 - Source data 6 - Hurwitz et al

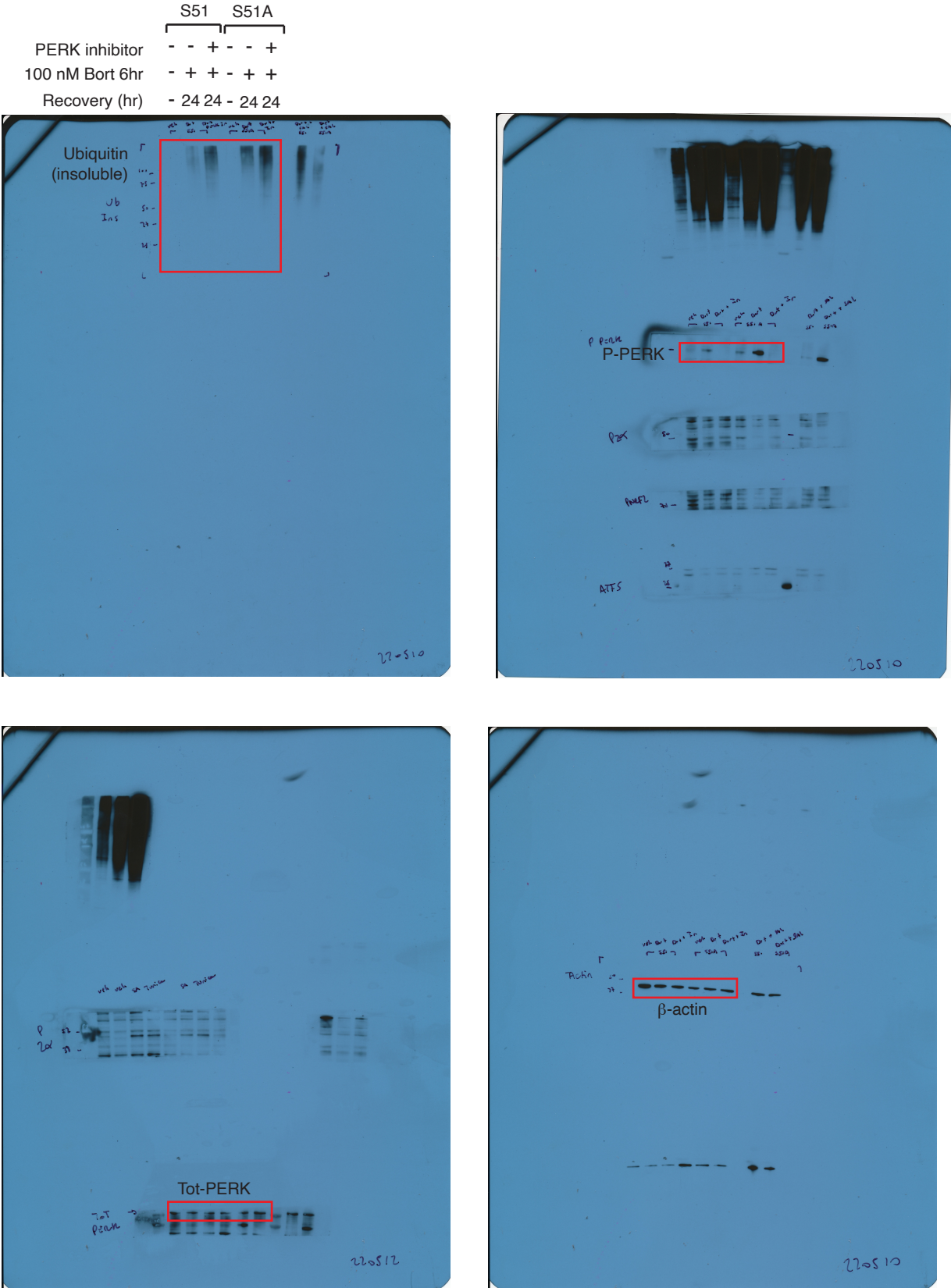

Supplement: Figure 2—figure supplement 3—source data 4. [file elife-77780-fig2-figsupp3-data4.pdf]

Figure 3 - Source data 1 - Hurwitz et al

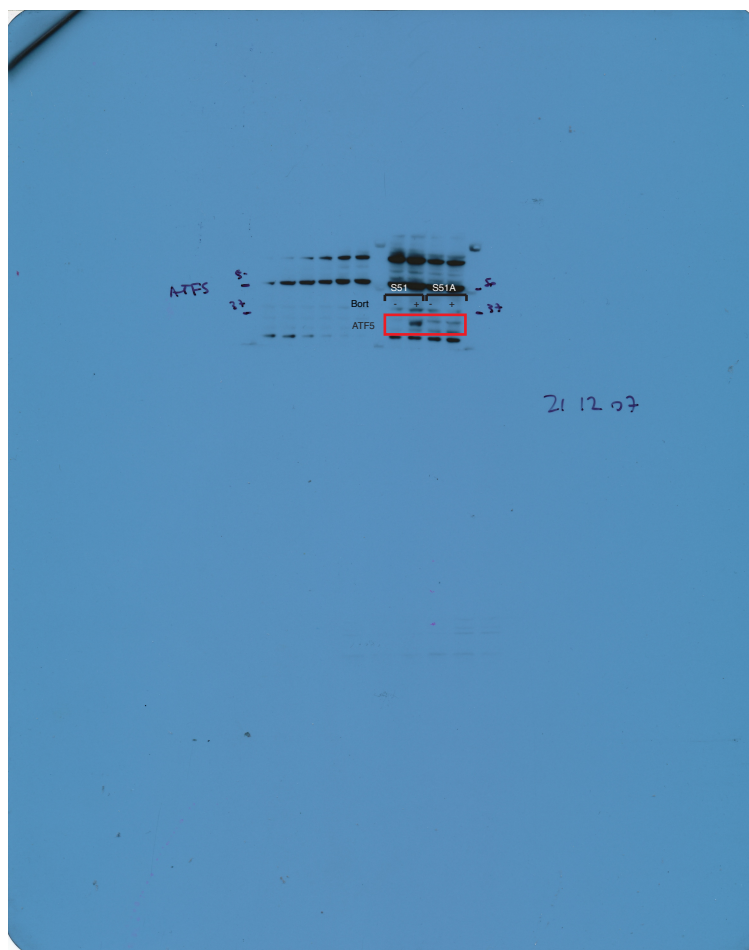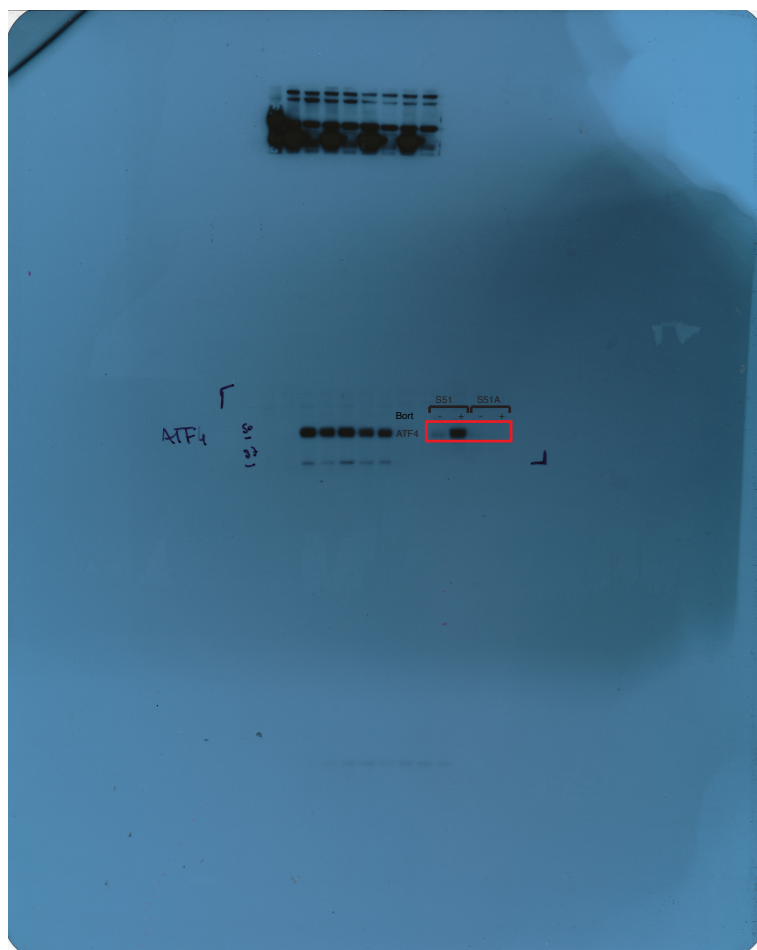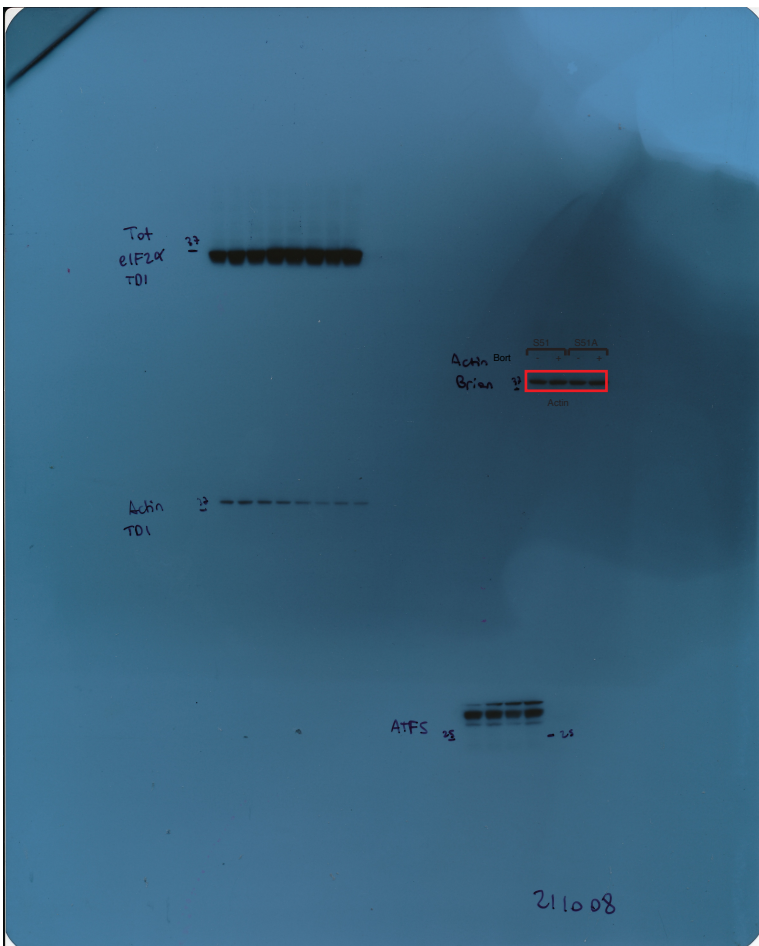

Supplement: Figure 3—source data 1. [file elife-77780-fig3-data1.pdf]

Figure 7 - Source data 1 - Hurwitz et al

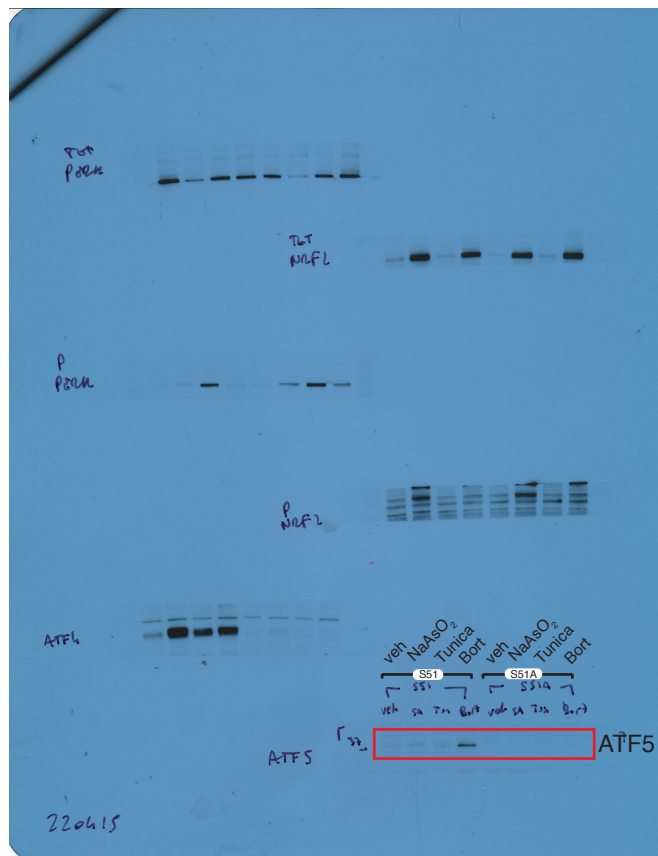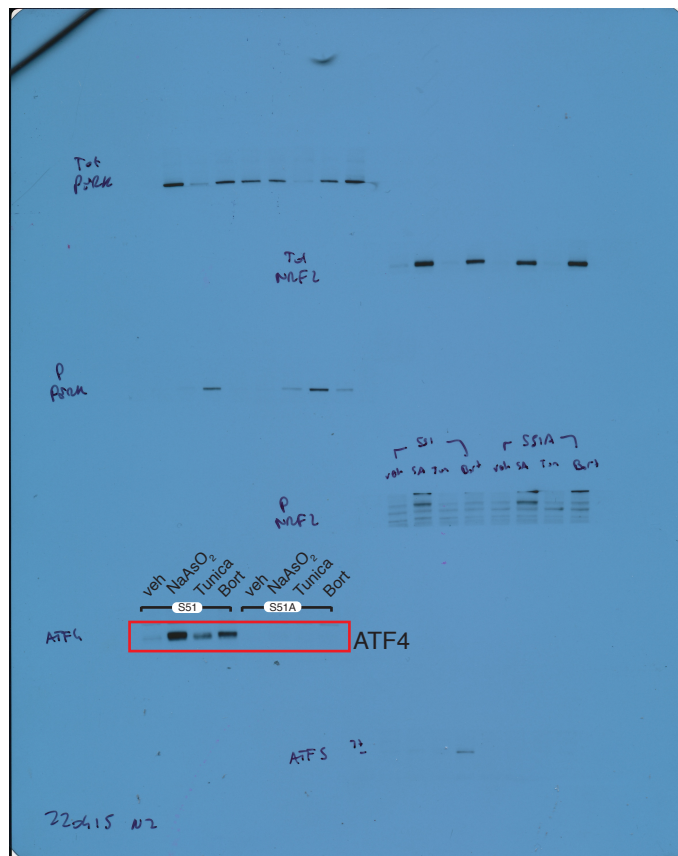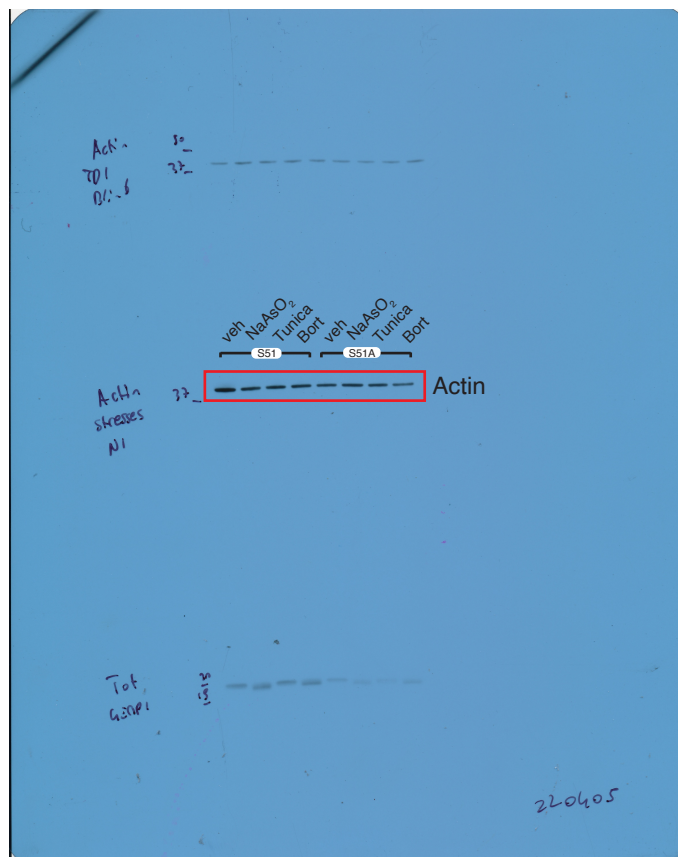

Supplement: Figure 7—source data 1. [file elife-77780-fig7-data1.pdf]

Figure 7 - Source data 2 - Hurwitz et al

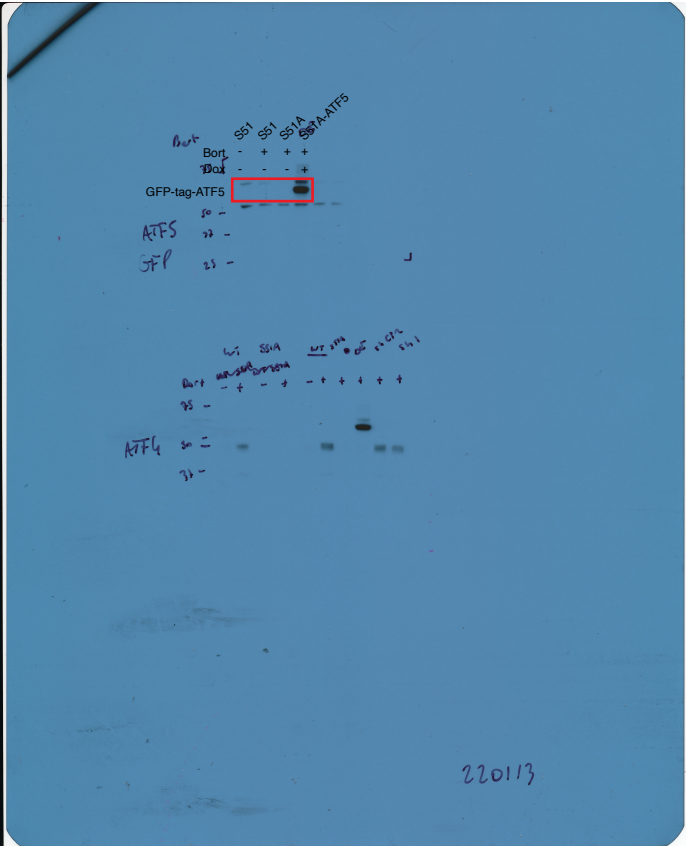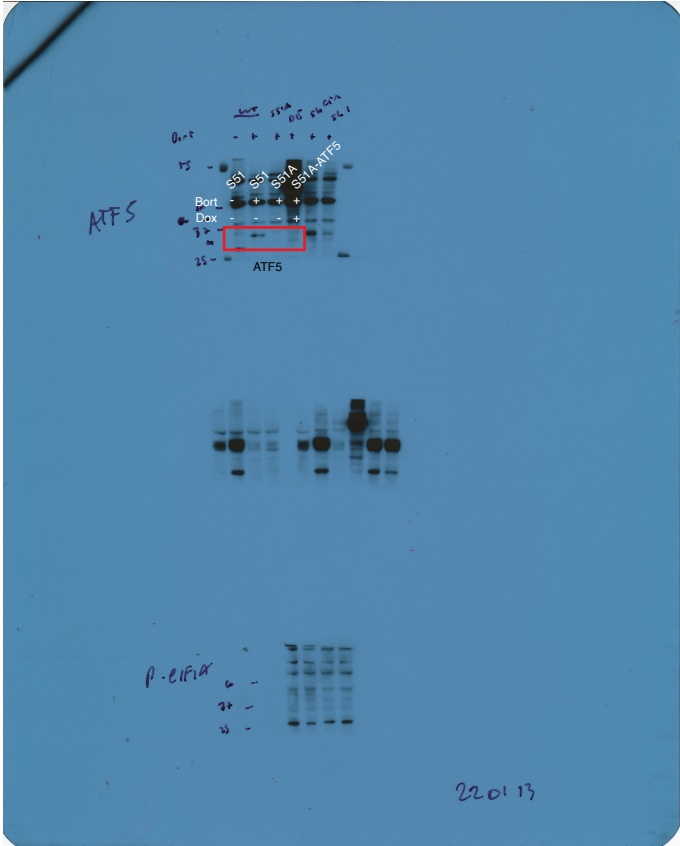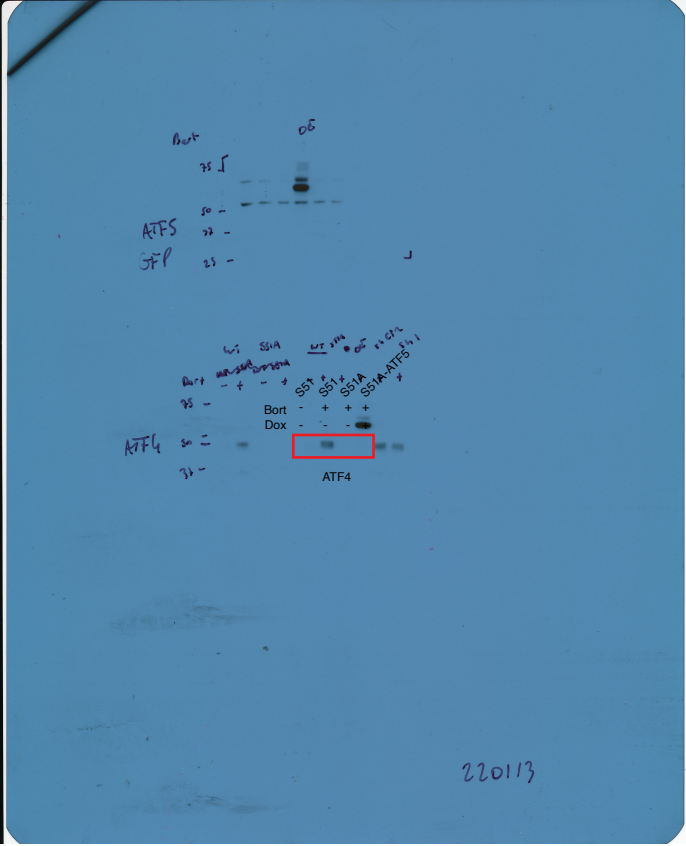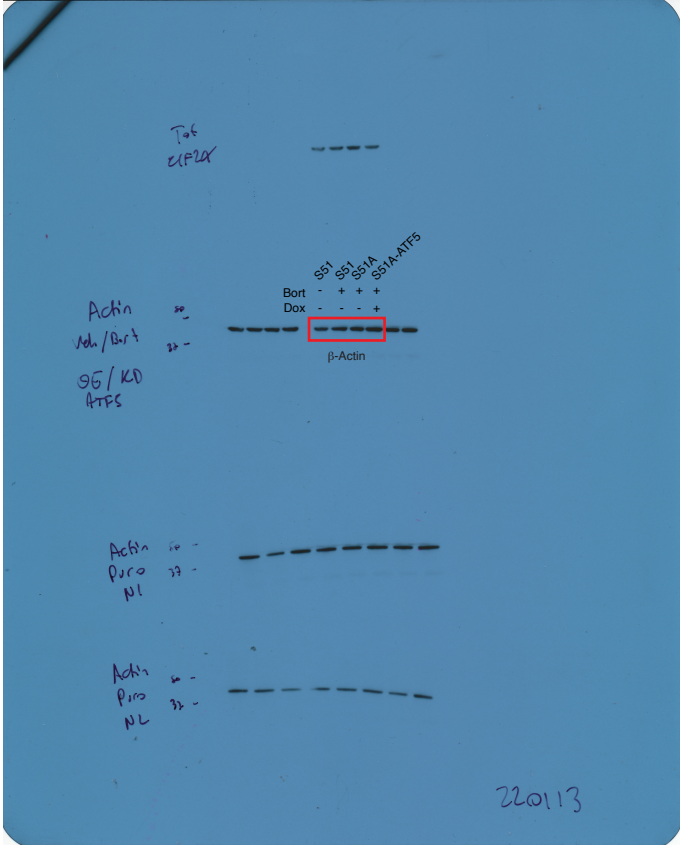

Supplement: Figure 7—source data 2. [file elife-77780-fig7-data2.pdf]

Figure 7- Source data 3 - Hurwitz et al

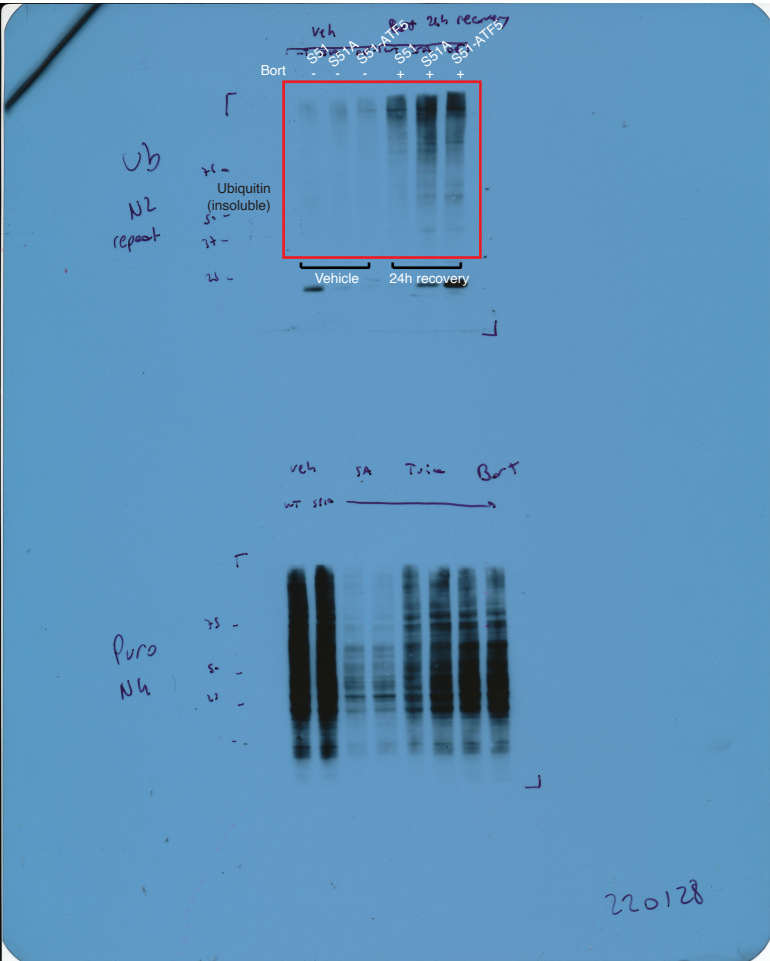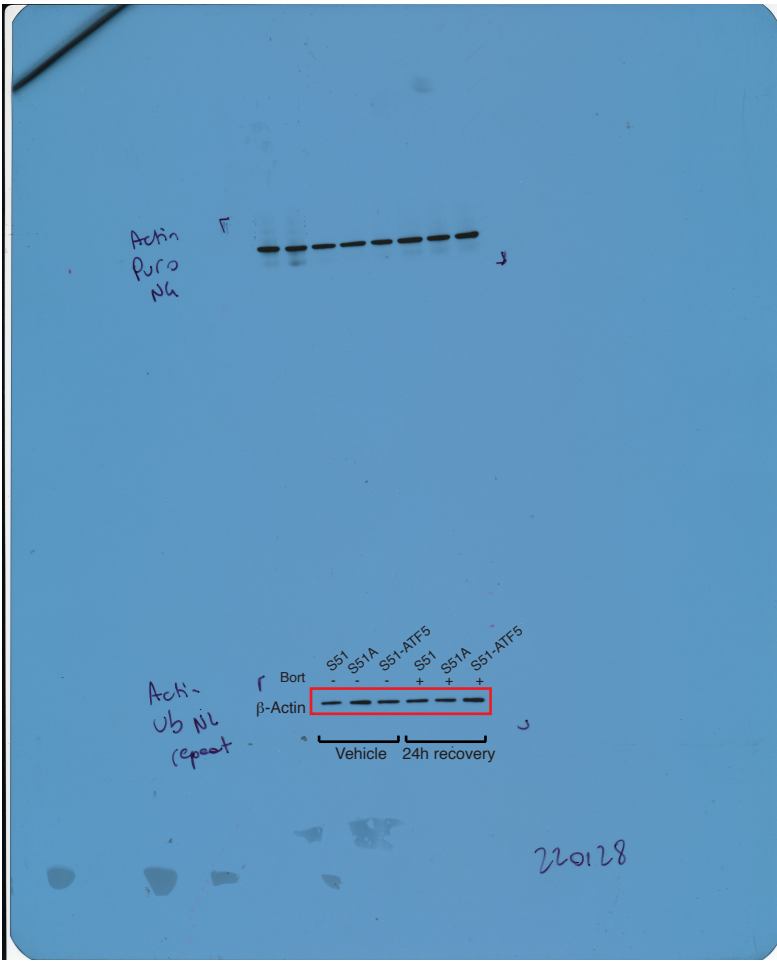

Supplement: Figure 7—source data 3. [file elife-77780-fig7-data3.pdf]

Figure 7 - Source data 4 - Hurwitz et al

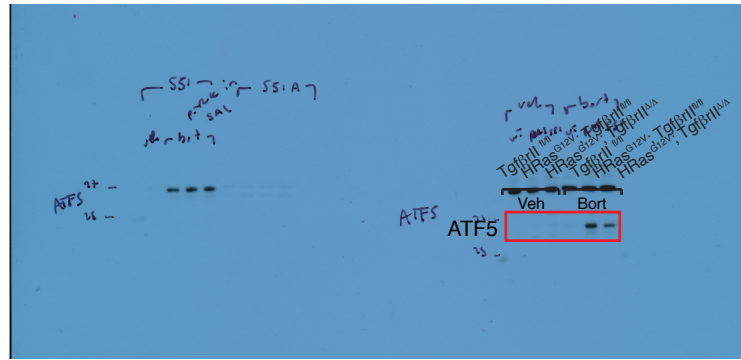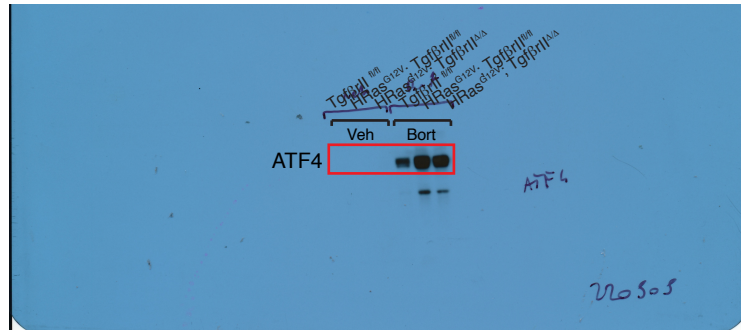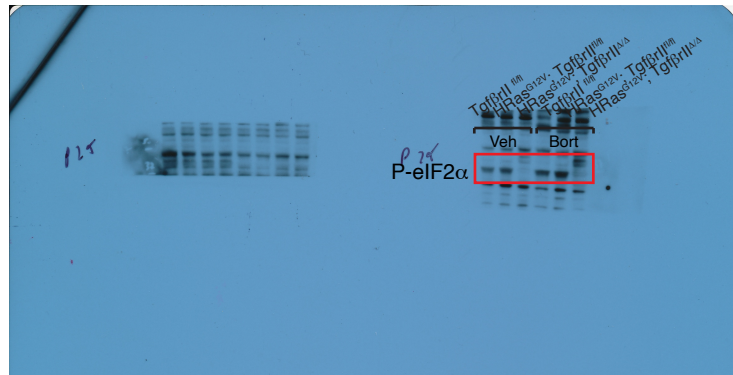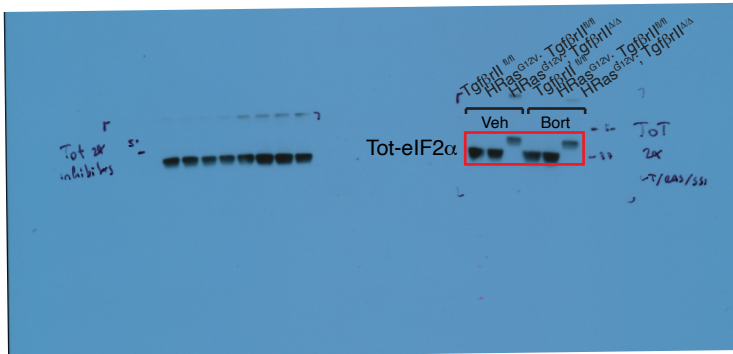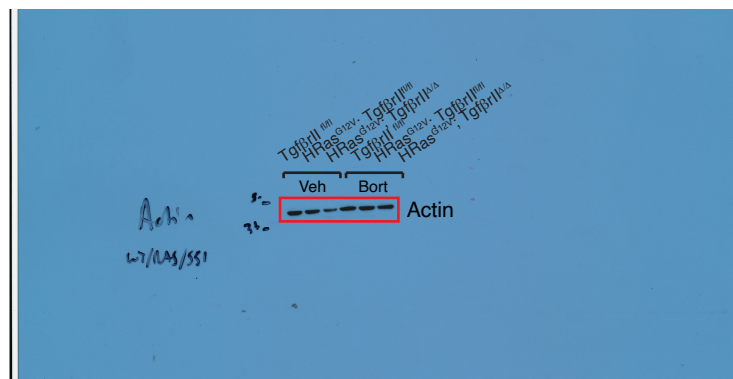

Supplement: Figure 7—source data 4. [file elife-77780-fig7-data4.pdf]
