# Supplementary material for: The integrated stress response remodels the microtubule-organizing center to clear unfolded proteins following proteotoxic stress
Source: eLife. 2022 Jun 27;11:e77780. doi: 10.7554/eLife.77780 (PMC9299849; doi:10.7554/eLife.77780)

Figure 2 - Source data 2 - Hurwitz et al

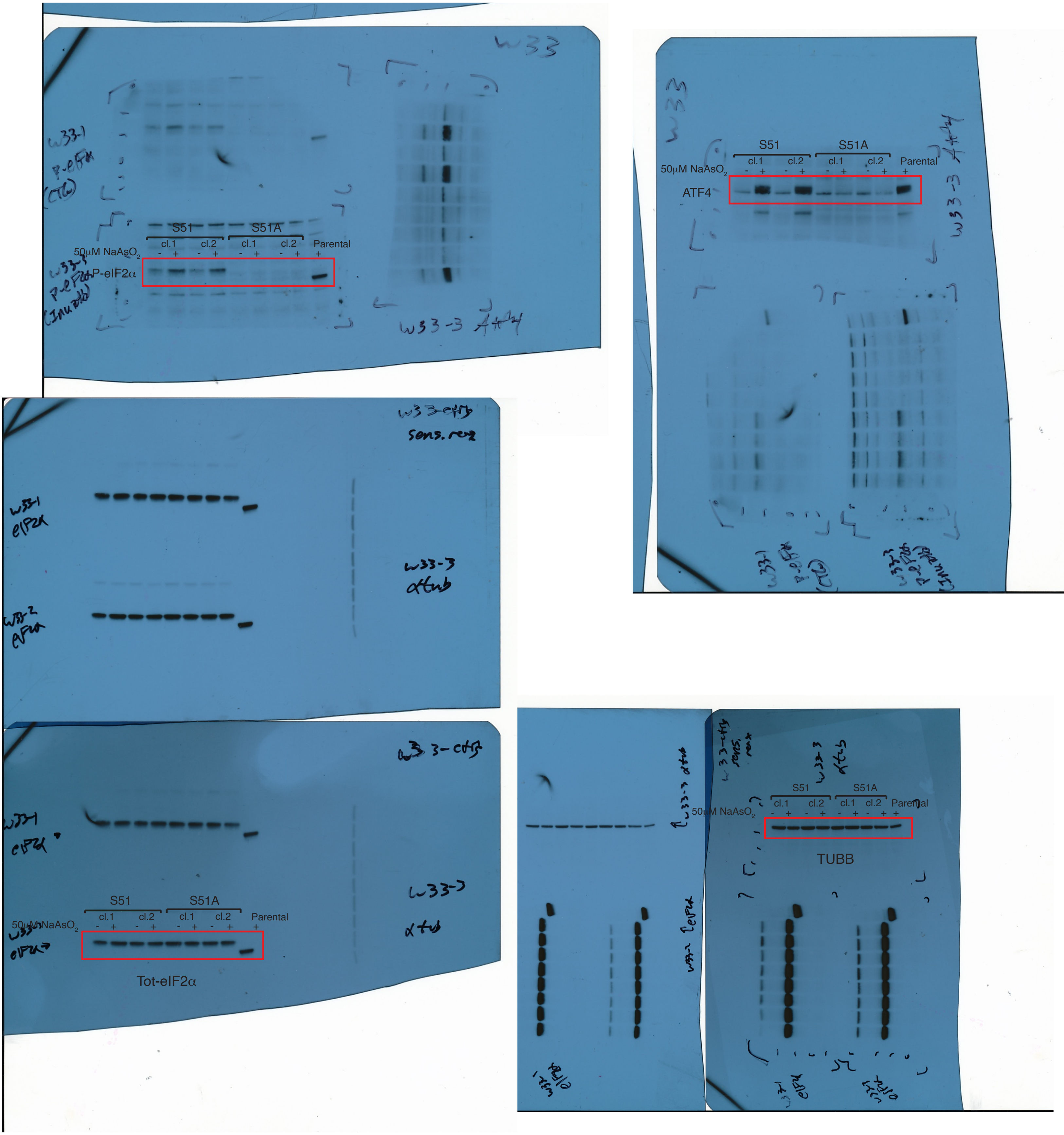

Supplement: Figure 2—figure supplement 1—source data 1. [file elife-77780-fig2-figsupp1-data1.pdf]
