# Supplementary material for: The integrated stress response remodels the microtubule-organizing center to clear unfolded proteins following proteotoxic stress
Source: eLife. 2022 Jun 27;11:e77780. doi: 10.7554/eLife.77780 (PMC9299849; doi:10.7554/eLife.77780)
Supplement: Transparent reporting form [file elife-77780-transrepform1.pdf]

Westbrook Centre
Milton Road
Cambridge CB4 1YG, UK

P 01223 855340
W elifesciences.org
T @elife

# *eLife's* transparent reporting form

We encourage authors to provide detailed information *within their submission* to facilitate the interpretation and replication of experiments. Authors can upload supporting documentation to indicate the use of appropriate reporting guidelines for health-related research (see EQUATOR Network), life science research (see the BioSharing Information Resource), or the ARRIVE guidelines for reporting work involving animal research. Where applicable, authors should refer to any relevant reporting standards documents in this form.

If you have any questions, please consult our Journal Policies and/or contact us: editorial@elifesciences.org.

**Sample-size estimation**

- You should state whether an appropriate sample size was computed when the study was being designed
- You should state the statistical method of sample size computation and any required assumptions
- If no explicit power analysis was used, you should describe how you decided what sample (replicate) size (number) to use

Please outline where this information can be found within the submission (e.g., sections or figure legends), or explain why this information doesn't apply to your submission:

Information calculating and/or justifying sample sizes for *in vivo* experiments can be found in the "Tumor allografting" subsection within the materials and methods section. Power analysis was not performed for *in vitro* experiments, rather minimum of 2 biological replicates was used for all experiments and either 3 or 4 technical replicates were performed for experiments with expected low and high variance, respectively.

Statistical method for each experiment is detailed in the corresponding figure legend.

**Replicates**

- You should report how often each experiment was performed
- You should include a definition of biological versus technical replication
- The data obtained should be provided and sufficient information should be provided to indicate the number of independent biological and/or technical replicates
- If you encountered any outliers, you should describe how these were handled
- Criteria for exclusion/inclusion of data should be clearly stated
- High-throughput sequence data should be uploaded before submission, with a private link for reviewers provided (these are available from both GEO and ArrayExpress)

Please outline where this information can be found within the submission (e.g., sections or figure legends), or explain why this information doesn't apply to your submission:

eLife Sciences Publications, Ltd is a limited liability non-profit non-stock corporation incorporated in the State of Delaware, USA, with company number 5030732, and is registered in the UK with company number FC030576 and branch number BR015634 at the address Westbrook Centre, Milton Road, Cambridge CB4 1YG.

Information describing biological and technical replicates can be found within figure legends for each individual experiment, in which biological replicates and technical replicates are listed.

Biological vs technical replicate:
- Biological replicate: experiment was repeated a different day, using cells at a different passage.
- Technical replicate: experiment was performed on the same day, using distinct cells plated at the same time.

- Ribo-seq and Total RNA seq datasets deposited to the Gene Expression Omnibus (GEO) repository with accession code GSE193945. Token for reviewers will be made available upon request.

eLife Sciences Publications, Ltd is a limited liability non-profit non-stock corporation incorporated in the State of Delaware, USA, with company number 5030732, and is registered in the UK with company number FC030576 and branch number BR015634 at the address Westbrook Centre, Milton Road, Cambridge CB4 1YG.

**Statistical reporting**

- Statistical analysis methods should be described and justified
- Raw data should be presented in figures whenever informative to do so (typically when N per group is less than 10)
- For each experiment, you should identify the statistical tests used, exact values of N, definitions of center, methods of multiple test correction, and dispersion and precision measures (e.g., mean, median, SD, SEM, confidence intervals; and, for the major substantive results, a measure of effect size (e.g., Pearson's r, Cohen's d)
- Report exact p-values wherever possible alongside the summary statistics and 95% confidence intervals. These should be reported for all key questions and not only when the p-value is less than 0.05.

Please outline where this information can be found within the submission (e.g., sections or figure legends), or explain why this information doesn't apply to your submission:

Choice of statistical test along with descriptions of the exact comparisons used can be found for each individual experiment within the figure legends. If no information on statistical testing is indicated, no statistical test was performed. Data from all replicates are visualized whenever possible.

(For large datasets, or papers with a very large number of statistical tests, you may upload a single table file with tests, Ns, etc., with reference to sections in the manuscript.)

**Group allocation**

- Indicate how samples were allocated into experimental groups (in the case of clinical studies, please specify allocation to treatment method); if randomization was used, please also state if restricted randomization was applied
- Indicate if masking was used during group allocation, data collection and/or data analysis

Please outline where this information can be found within the submission (e.g., sections or figure legends), or explain why this information doesn't apply to your submission:

Tumor bearing mice used for bortezomib treatment experiments were randomly allocated to vehicle or bortezomib treatment. Blinding following allocation was not performed for this experiment. Group allocation does not apply to other experiments in this study.

**Additional data files ("source data")**

- We encourage you to upload relevant additional data files, such as numerical data that are represented as a graph in a figure, or as a summary table
- Where provided, these should be in the most useful format, and they can be uploaded as "Source data" files linked to a main figure or table
- Include model definition files including the full list of parameters used
- Include code used for data analysis (e.g., R, MatLab)
- Avoid stating that data files are "available upon request"

Please indicate the figures or tables for which source data files have been provided:

eLife Sciences Publications, Ltd is a limited liability non-profit non-stock corporation incorporated in the State of Delaware, USA, with company number 5030732, and is registered in the UK with company number FC030576 and branch number BR015634 at the address Westbrook Centre, Milton Road, Cambridge CB4 1YG.

Source data for each experiment yielding numerical data will be uploaded as .csv files with manuscript publication (see Source data 11).

Raw immunoblots and original gel files for each figures are uploaded as Source data (Source data 1-10).

Raw data and count files for ribosome profiling experiments have been deposited into the Gene Expression Omnibus (GEO) with accession code: GSE193945. Raw count files for ribosome profiling and RPF are uploaded as Source data 12.
